# Association between admission-blood-glucose-to-albumin ratio and clinical outcomes in patients with ST-elevation myocardial infarction undergoing percutaneous coronary intervention

**DOI:** 10.3389/fcvm.2023.1132685

**Published:** 2023-09-07

**Authors:** Cien Zhen, Wei Chen, Weikun Chen, Hualin Fan, Zijing Lin, Lihuan Zeng, Zehuo Lin, Weibin He, Yu Li, Shimin Peng, Lin Zeng, Chongyang Duan, Ning Tan, Yuanhui Liu, Pengcheng He

**Affiliations:** ^1^ Department of Cardiology, School of Medicine, South China University of Technology, Guangzhou, China; ^2^ Department of Cardiology, Guangdong Provincial People's Hospital (Guangdong Academy of Medical Sciences), Southern Medical University, Guangzhou, China; ^3^ Department of Cardiology, Guangdong Provincial Key Laboratory of Coronary Heart Disease Prevention, Guangdong Provincial People's Hospital (Guangdong Academy of Medical Sciences), Guangdong Cardiovascular Institute, Guangzhou, China; ^4^ Department of Cardiology, Fujian Provincial Clinical College of Fujian Medical University, Fujian Provincial Hospital, Fujian Institute of Cardiovascular Disease, Fuzhou, China; ^5^ The Second School of Clinical Medicine, Southern Medical University, Guangzhou, China; ^6^ Department of Cardiology, Guangdong Provincial People's Hospital's Nanhai Hospital, The Second Hospital of Nanhai District Foshan City, Foshan, China; ^7^ Shantou University Medical College, Shantou, China; ^8^ Department of Biostatistics, School of Public Health, Southern Medical University, Guangzhou, China

**Keywords:** percutaneous coronary intervention, admission blood glucose, albumin, predictor, ST-segment elevation myocardial infarction

## Abstract

**Introduction:**

It is unclear whether admission-blood-glucose-to-albumin ratio (AAR) predicts adverse clinical outcomes in patients with ST-segment elevation myocardial infarction (STEMI) who are treated with percutaneous coronary intervention (PCI). Here, we performed a observational study to explore the predictive value of AAR on clinical outcomes.

**Methods:**

Patients diagnosed with STEMI who underwent PCI between January 2010 and February 2020 were enrolled in the study. The patients were classified into three groups according to AAR tertile. The primary outcome was in-hospital all-cause mortality, and the secondary outcomes were in-hospital major adverse cardiac events (MACEs), as well as all-cause mortality and MACEs during follow-up. Logistic regression, Kaplan–Meier analysis, and Cox proportional hazard regression were the primary analyses used to estimate outcomes.

**Results:**

Among the 3,224 enrolled patients, there were 130 cases of in-hospital all-cause mortality (3.9%) and 181 patients (5.4%) experienced MACEs. After adjustment for covariates, multivariate analysis demonstrated that an increase in AAR was associated with an increased risk of in-hospital all-cause mortality [adjusted odds ratio (OR): 2.72, 95% CI: 1.47–5.03, *P* = 0.001] and MACEs (adjusted OR: 1.91, 95% CI: 1.18–3.10, *P* = 0.009), as well as long-term all-cause mortality [adjusted hazard ratio (HR): 1.64, 95% CI: 1.19–2.28, *P* = 0.003] and MACEs (adjusted HR: 1.58, 95% CI: 1.16–2.14, *P *= 0.003). Receiver operating characteristic (ROC) curve analysis indicated that AAR was an accurate predictor of in-hospital all-cause mortality (AUC = 0.718, 95% CI: 0.675–0.761) and MACEs (AUC = 0.672, 95% CI: 0.631–0.712).

**Discussion:**

AAR is a novel and convenient independent predictor of all-cause mortality and MACEs, both in-hospital and long-term, for STEMI patients receiving PCI.

## 1. Introduction

ST-segment elevation myocardial infarction (STEMI) remains a major cause of mortality worldwide, despite the common use of standardized interventional and medical treatments (1, 2). Early risk evaluation of patients during hospitalization is useful in making clinical decisions and selecting the optimal treatment. In recent years, risk assessment of STEMI patients has commonly been performed using the Global Registry of Acute Coronary Events (GRACE) risk score, which includes medical history, some clinical symptoms and signs, cardiac biomarkers, and electrocardiographic changes (2). Nonetheless, the GRACE risk score is complicated for use in the emergency department of primary hospitals, particularly in developing countries. Therefore, simple and easily accessible indicators with high prognostic value for outcomes in such patients are required.

Blood glucose and albumin levels at admission are rapidly and easily accessible biomarkers for use in the emergency room. An increased blood glucose level at admission indicates diabetes mellitus (DM), which may accelerate the progression of STEMI (3). Accordingly, some studies have shown that patients with STEMI who have a higher admission blood glucose level are at an increased risk of adverse events (4–8). Recent studies have indicated that admission blood glucose level may also be increased in stress-induced hyperglycemia (SIH) due to the response of the sympathetic nervous system to physiological and emotional stress, leading to an increased risk of acute cardiovascular diseases (9). Therefore, admission blood glucose level is a valuable indicator to evaluate the prognosis of individuals with cardiovascular disease.

Although traditionally considered to be an indicator of malnutrition, albumin (10, 11) is a multifunctional protein associated with DM, inflammation, and thrombosis, which are risk factors for or triggers of STEMI or its progression (12). In addition, recent studies have shown that a decrease in serum albumin is linked to an increase in cardiovascular mortality (13–16). An indicator consisting of a combination of albumin and admission blood glucose levels may guide the need for malnutrition treatment and blood glucose control, as well as indicating the occurrence of a thrombo-inflammatory reaction during STEMI and subsequent percutaneous coronary intervention (PCI) treatment. Therefore, we hypothesized that the admission-blood-glucose-to-albumin ratio (AAR), a novel composite index of two biomarkers, may predict adverse clinical outcomes for STEMI patients receiving PCI. In this study, the predictive value of AAR was explored in a study with a large sample size.

## 2. Methods

### 2.1. Study population

Between January 2010 and February 2020, we enrolled STEMI patients undergoing PCI at the Guangdong Provincial People's Hospital. The definition of STEMI was determined in accordance with the guidelines of the European Society of Cardiology (2, 17). We excluded any patients: (1) who had a diagnosis of non-ST-elevation myocardial infarction (NSTEMI); (2) who did not undergo PCI treatment; (3) for whom no admission laboratory parameters were available, or for whom variables needed to calculate the ratio were missing; or (4) who had active carcinoma or acute/chronic inflammatory diseases. This study was conducted according to the guidelines laid down in the Declaration of Helsinki, and all procedures involving human subjects/patients were approved by the research ethics committee of Guangdong Provincial People's Hospital (No. GDREC2016378H). Written informed consent was obtained from all participants.

### 2.2. Procedure and data collection

All patients were treated in accordance with the hospital protocol and the European Society of Cardiology guidelines for STEMI patients (2, 17). Within 24 h of admission, before PCI, blood samples were obtained to measure the relevant laboratory parameters, including admission blood glucose and serum albumin levels. Before PCI, all eligible patients were prescribed aspirin (300 mg) and clopidogrel (300 or 600 mg) or ticagrelor (180 mg). Following the recommendations for catheter procedures (18), coronary angiography and PCI were performed by experienced interventional cardiologists, who made all decisions relating to the procedures, including the choice of vessel access, the selection of stents, the volume of contrast, and so on. After PCI, patients received dual antiplatelet therapy, including aspirin (100 mg/day) combined with clopidogrel (75 mg/day) or ticagrelor (90 mg twice/day). Other medications were prescribed by the patients’ physicians according to standard regimens. Clinical information on demographic characteristics and other relevant data on the enrolled patients were extracted from the hospital electronic database by trained researchers. Follow-up data were collected by trained nurses through telephone tracking or outpatient visits.

### 2.3. Outcomes and definitions

In-hospital all-cause mortality was the primary outcome. The secondary outcomes were in-hospital major adverse cardiac events (MACEs), as well as all-cause mortality and MACEs during follow-up. In-hospital MACEs were defined as the combined endpoint of all-cause mortality, stroke, target vessel revascularization, and recurrent myocardial infarction during hospitalization. MACEs during follow-up were defined as the combined endpoint of follow-up all-cause mortality or stroke.

### 2.4. Statistical analyses

AAR was calculated as [admission blood glucose (mmol/L) × 18/albumin (g/L)] or [admission blood glucose (mg/dl)/albumin (g/L)] using the same blood samples obtained at admission. The value of AAR was calculated for every patient according to the formula. AAR values were stratified into tertiles, according to which patients were divided into three groups: T1 (*n* = 1,074; AAR < 3.35), T2 (*n* = 1,076; 3.35 ≤ AAR < 4.56), and T3 (*n* = 1,074; AAR ≥ 4.56). Categorical variables are reported in the form of numbers (percentages). In order to compare the distributions of categorical variables, Chi-square tests or Fisher's exact tests were employed. For continuous variables, normally distributed data are reported in the form of means ± standard deviation (SD), while non-normally distributed data are reported in the form of medians (Q25–Q75). Normally distributed continuous variables were compared using one-way ANOVA; otherwise, the Kruskal–Wallis test was performed. To calculate odds ratios (ORs) and 95% confidence intervals (CIs) for in-hospital mortality and MACEs according to AAR, we performed univariate and multivariate logistic regression analyses. Model 1 was adjusted for, age, sex, smoking status, prior stroke, chronic obstructive pulmonary disease, diabetes mellitus (DM), hypertension, prior myocardial infarction, prior PCI, anemia, Killip class ≥II, estimated glomerular filtration rate (eGFR), aspirin, glycoprotein IIb/IIIa inhibitors, multiple lesions, and transradial access. Model 2 was adjusted for alanine aminotransferase (ALT) in addition to the covariates adjusted for in Model 1; Model 3 was adjusted for insulin in addition to the covariates adjusted for in Model 1; and Model 4 was adjusted for heart rate and prior cardiac arrest in addition to the covariates adjusted for in Model 1. These models were used to perform univariate and multivariate logistic regression analyses. To determine how well AAR predicts in-hospital mortality and MACEs, receiver operating characteristic (ROC) curves were constructed and the area under the ROC curve (AUC) was subsequently calculated. The AUC value can be used to evaluate the efficacy of the predictor (AUC <0.6, poor discrimination; 0.6–0.75, good discrimination; >0.75, excellent discrimination) (19). The optimal cutoff value was determined by the maximum Youden index (Youden = sensitivity + specificity–1). To assess the prognostic value of AAR in patients with and without DM, ROC curves were plotted to determine the optimal cutoff value for AAR as a predictor of MACEs in each of the two groups. In addition, nomograms for the predictive value of in-hospital all-cause mortality and MACEs were constructed. To assess for non-linearity in the relationship between AAR as a continuous variable and in-hospital death or MACEs, we fitted restricted cubic spline models to identify a non-linear relationship. Kaplan–Meier analysis was conducted to visualize several survival curves illustrating follow-up risks of mortality and MACEs in different AAR groups. The effect of AAR on long-term mortality and MACEs was assessed using univariate and multivariate Cox proportional hazards regression analyses in Models 1–4. Log-rank tests were used to compare survival curves between groups. A *P*-value of 0.05 or below was considered to represent statistical significance. SAS version 9.4 (SAS Institute, Cary, NC, USA) was used to perform the statistical analyses.

## 3. Results

### 3.1. Baseline clinical characteristics according to AAR tertiles

**Figure 1:**
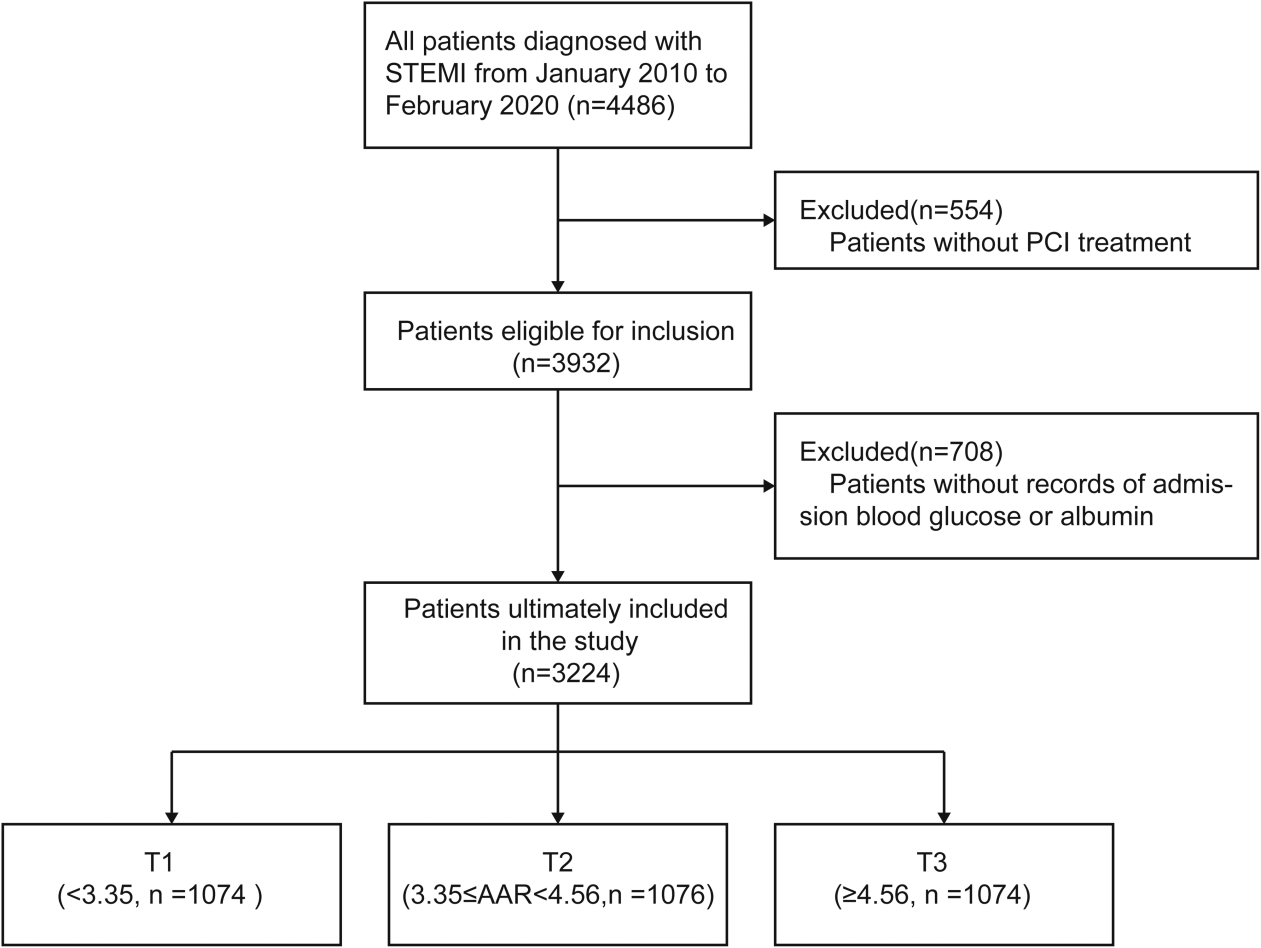
Flow chart of the study participants.

A flow chart of the study is presented in Figure 1. Patients were divided into three groups according to AAR tertile. The mean AAR value across all patients was 4.58 ± 2.38, and the average age was 62.10 ± 12.24 years. The baseline clinical characteristics of the three groups are presented in Table 1. Compared to the other groups, patients in the T3 group were older, had a higher Killip class, stayed in hospital for longer, had lower systolic and diastolic blood pressure, and were more likely to have been treated with prior PCI and to have been diagnosed with hypertension, anemia, DM, and stroke. In addition, a higher AAR was associated with higher hemoglobin A1c (HbA1c) and serum creatinine levels, as well as lower low-density lipoprotein cholesterol, total cholesterol, hemoglobin, left ventricular ejection fraction, and eGFR.

**Table 1:** Patients’ baseline characteristics by AAR tertile.

Variables	T1 (*n* = 1,074)	T2 (*n* = 1,076)	T3 (*n* = 1,074)	*P*-value
Age				
Age ≥ 65 years, *n* (%)	342 (31.8)	506 (47.0)	559 (52.1)	<0.001
Mean ± SD (years)	58.81 ± 12.27	62.94 ± 12.15	64.54 ± 11.57	<0.001
Sex, *n* (%)				
Male	955 (88.9)	902 (83.8)	811 (75.5)	<0.001
Female	119 (11.1)	174 (16.2)	263 (24.5)	<0.001
Smoker, *n* (%)	534 (49.8)	437 (40.6)	378 (35.2)	<0.001
Comorbidities, *n* (%)				
Hypertension	482 (44.9)	548 (50.9)	619 (57.6)	<0.001
Diabetes mellitus	81 (7.5)	163 (15.1)	686 (63.9)	<0.001
Hyperlipidemia	169 (15.7)	120 (11.2)	111 (10.3)	<0.001
COPD	22 (2.0)	26 (2.4)	23 (2.1)	0.833
Anemia	258 (24.1)	353 (32.9)	422 (39.3)	<0.001
Prior myocardial infarction	284 (26.4)	196 (18.2)	241 (22.4)	<0.001
Prior PCI	80 (7.4)	92 (8.6)	117 (10.9)	0.017
Prior stroke	51 (4.7)	61 (5.7)	111 (10.3)	<0.001
Prior atrial fibrillation	29 (2.7)	33 (3.1)	39 (3.6)	0.459
Prior cardiac arrest	52 (4.8)	61 (5.7)	75 (7.0)	0.102
Heart rate (bpm)	77.81 ± 14.16	80.01 ± 15.64	83.66 ± 18.41	<0.001
SBP (mmHg)	123.90 ± 20.28	121.22 ± 21.69	120.48 ± 22.86	<0.001
DBP (mmHg)	75.88 ± 13.33	73.77 ± 13.44	72.40 ± 13.40	<0.001
Killip class ≥ II	223 (20.8)	321 (29.9)	438 (40.8)	<0.001
Examination results				
Total cholesterol (mmol/L)	4.90 (4.24–5.70)	4.70 (4.02–5.47)	4.61 (3.83–5.50)	<0.001
LDL-C (mmol/L)	3.27 (2.70–3.86)	3.05 (2.49–3.68)	2.97 (2.35–3.71)	<0.001
HbA1c (%)	5.80 (5.50–6.10)	6.00 (5.60–6.40)	7.40 (6.30–9.20)	<0.001
Hemoglobin (g/L)	139.60 (128.95–150.00)	134.70 (124.10–146.00)	131.90 (117.95–145.00)	<0.001
Admission blood glucose (mmol/L)	5.76 (5.26–6.33)	7.41 (6.77–8.15)	11.56 (9.60–15.00)	<0.001
Serum albumin (g/L)	37.00 (34.40–39.30)	34.75 (32.33–37.10)	33.30 (30.10–36.40)	<0.001
eGFR (ml/min/1.73m^2^)	89.97 (73.08–108.04)	85.42 (69.79–102.48)	75.86 (52.68–96.89)	<0.001
Serum creatinine (µmol/L)	83.10 (71.61–98.13)	85.75 (72.49–99.89)	91.94 (75.14–121.11)	<0.001
ALT (U/L)	40.00 (27.00–57.68)	45.00 (29.00–68.00)	42.00 (28.00–70.00)	<0.001
LVEF (%)	55.00 (46.00–61.00)	53.00 (43.00–60.00)	50.00 (40.00–59.00)	<0.001
Medication, *n* (%)				
Aspirin	1,065 (99.2)	1,066 (99.1)	1,051 (97.9)	0.012
Clopidogrel	931 (86.7)	965 (89.9)	979 (91.2)	0.002
Glycoprotein IIb/IIIa inhibitors	363 (33.9)	420 (39.2)	391 (36.4)	0.041
Statins	1,053 (98.0)	1,059 (98.5%)	1,045 (97.4)	0.179
ACEI/ARB	889 (82.8)	872 (81.0)	842 (78.4)	0.035
CCB	101 (9.4)	90 (8.4)	125 (11.7)	0.032
Beta-blockers	899 (83.7)	885 (82.3)	863 (80.4)	0.138
Insulin	16 (1.5)	58 (5.4)	434 (40.4)	<0.001
Procedures for PCI				
Transradial access, *n* (%)	988 (92.0)	921 (85.8)	847 (79.0)	<0.001
Transfemoral access, *n* (%)	86 (8.0)	153 (14.2)	225 (21.0)	<0.001
No. of stents	1.53 ± 3.13	1.45 ± 1.28	1.52 ± 1.41	0.659
Contrast volume ≥100 ml, *n* (%)	789 (77.0)	844 (82.3)	801 (78.3)	0.009
Multi-lesion, *n* (%)	730 (68.0)	790 (73.4)	820 (76.4)	<0.001
Time interval from admission to PCI (days)	0.00 (0.00–1.00)	0.00 (0.00–1.00)	0.00 (0.00–1.00)	0.090
Length of hospital stay (days)	6.00 (5.00–8.00)	7.00 (5.00–9.00)	7.00 (6.00–11.00)	<0.001

Values are given as *n* (%), mean ± SD, or median (Q25–Q75).

AAR, admission-blood-glucose-to-albumin ratio; ACEI/ARB, angiotensin-converting enzyme inhibitors/angiotensin receptor blockers; CCB, calcium channel blockers; COPD, chronic obstructive pulmonary disease; DBP, diastolic blood pressure; eGFR, estimated glomerular filtration rate; HbA1c, hemoglobin A1c; LDL-C, low-density lipoprotein cholesterol; LVEF, left ventricular ejection fraction; PCI, percutaneous coronary intervention; SBP, systolic blood pressure; SD, standard deviation.

### 3.2. In-hospital clinical outcomes

In total, 130 patients (3.9%) died in hospital and 181 (5.4%) experienced in-hospital MACEs. Moreover, significantly higher rates of in-hospital all-cause mortality and MACEs were reported in the T3 group compared to the T1 group (8.0% vs. 1.5% and 9.9% vs. 3.0%, respectively; *P* < 0.001).

Among these STEMI patients after PCI, the continuous variable of AAR was positively associated with risk of in-hospital all-cause mortality (OR: 1.22, 95% CI: 1.16–1.27, *P* = 0.000) and MACEs (OR: 1.19, 95% CI: 1.14–1.24, *P* = 0.000) in univariate logistic regression analyses. After adjusting for confounders in the multivariate logistic regression analysis, the results showed that a higher risk of in-hospital all-cause mortality (adjusted OR: 1.14, 95% CI: 1.07–1.22, *P* = 0.000) and MACEs (adjusted OR: 1.11, 95% CI: 1.05–1.18, *P* = 0.001) (Supplementary Table S1, Model 1) was independently predicted by AAR, indicating that AAR was an independent predictor of these outcomes. When AAR was examined as a categorical variable, univariate logistic regression analysis revealed that the T3 group was at an elevated risk of unfavorable outcomes in terms of in-hospital all-cause mortality (OR: 5.75, 95% CI: 3.35–9.87, *P* = 0.000) and MACEs (OR: 3.57, 95% CI: 2.38–5.35, *P* = 0.000), but not the T2 group (OR: 1.77, 95% CI: 0.95–3.28, *P* = 0.072; OR: 1.36, 95% CI: 0.85–2.16, *P* = 0.201, respectively). Furthermore, the results of the multivariate logistic regression analysis revealed that AAR level was substantially associated with the adjusted risk of in-hospital all-cause mortality, with higher adjusted risk in the T3 group (adjusted OR: 2.72, 95% CI: 1.47–5.03, *P* = 0.001) and MACEs (adjusted OR: 1.91, 95% CI: 1.18–3.10, *P* = 0.009) as compared to the T1 group (Supplementary Table S2, Model 1). Furthermore, when considering the effects of factors potentially associated with albumin or blood glucose and several important determinants of prognosis, these findings of the logistic regression analyses were not materially affected in additional models that further adjusted for ALT (Supplementary Tables S1 and S2, Model 2), insulin (Supplementary Tables S1 and S2, Model 3), heart rate, and prior cardiac arrest (Supplementary Tables S1 and S2, Model 4).

### 3.3. Predictive value of AAR

**Figure 2:**
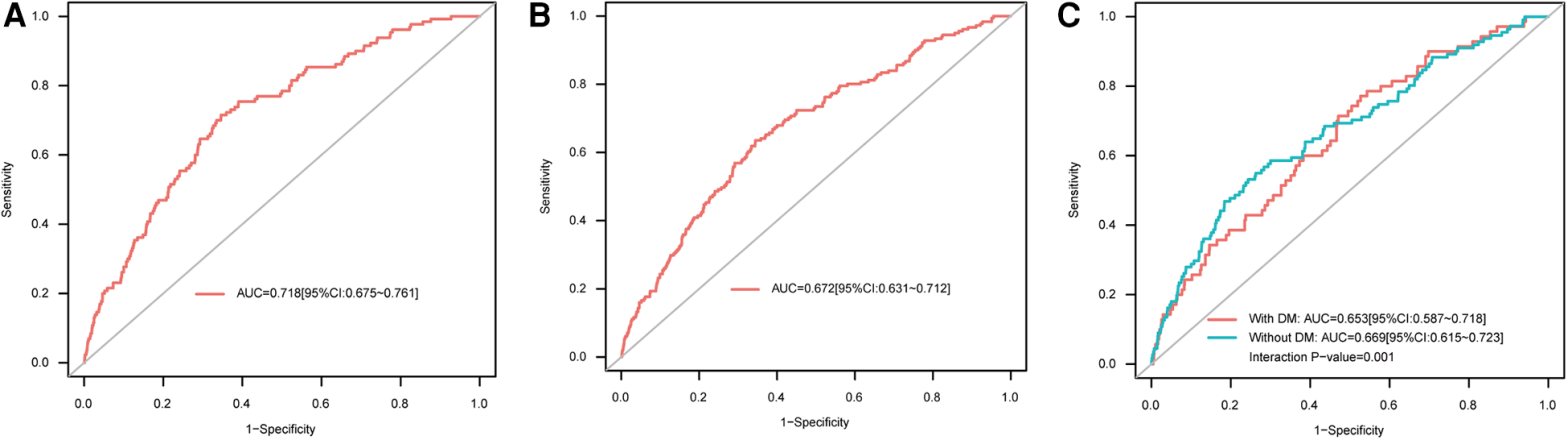
ROC curves for AAR as a predictor of (A) in-hospital all-cause mortality, (B) in-hospital MACEs, and (C) in-hospital all-cause mortality in the DM subgroup. AAR, admission-blood-glucose-to-albumin ratio; AUC, area under the ROC curve; DM, diabetes mellitus; MACEs, major adverse cardiac events; ROC, receiver operating characteristic.

ROC curve analysis showed that AAR could predict in-hospital all-cause mortality (AUC = 0.718, 95% CI: 0.675–0.761) (Figure 2A) as well as MACEs (AUC = 0.672, 95% CI: 0.631–0.712) (Figure 2B). The optimal cutoff value for AAR was 4.429, as calculated on the basis of the Youden index. Interestingly, according to ROC curve analysis in the DM subgroup, the predictive value of AAR for in-hospital all-cause mortality was different for patients with and without DM (with-DM group: AUC = 0.653, 95% CI: 0.587–0.718; without-DM group: AUC = 0.669, 95% CI: 0.615–0.723; interaction *P* = 0.001) (Figure 2C). Furthermore, it is necessary to account for HbA1c, one of the indicators of DM and long-term glycemic control, in evaluating the prognostic value of AAR. However, additional ROC analyses for HbA1c subgroups revealed that there was no significant difference between HbA1c subgroups for either in-hospital all-cause mortality (HbA1c ≤6.1 subgroup: AUC = 0.723, 95% CI: 0.662–0.783; HbA1c >6.1 subgroup: AUC = 0.749, 95% CI: 0.691–0.808; *P* for comparison = 0.535) or in-hospital MACEs (HbA1c ≤6.1 subgroup: AUC = 0.666, 95% CI: 0.609–0.722; HbA1c >6.1 subgroup: AUC = 0.704, 95% CI: 0.648–0.761; *P* for comparison = 0.343).

Several other factors should be considered in the evaluation of the predictive value of AAR. In order to compare AAR with two individual indices (admission blood glucose and albumin), ROC curve analyses for these three indicators were conducted (Supplementary Figure S1). These analyses showed that, for both in-hospital all-cause mortality and in-hospital MACEs, the predictive value of AAR was significantly greater than that of admission blood glucose (AUC for mortality = 0.664, 95% CI: 0.671–0.712, *P* for comparison with AAR <0.0001; AUC for MACEs = 0.633, 95% CI: 0.591–0.676, *P* for comparison with AAR <0.0001), while the AUC for AAR was marginally higher than the AUC for albumin (AUC for mortality = 0.701, 95% CI: 0.655–0.747, *P* for comparison with AAR = 0.508; AUC for MACEs = 0.655, 95% CI: 0.614–0.696, *P* for comparison with AAR = 0.466). In addition, time heterogeneity should be considered. Specifically, given the fluctuations in glucose level that occur over the 24 h of each day (20, 21), we wondered whether the effect of postprandial blood glucose had an impact on the prognostic value of AAR. To test this, we divided patients into three groups according to the time period in which the admission measurements were taken: 06:00–12:00 (morning), 12:00–18:00 (afternoon), and 18:00–06:00 (nighttime); these groups consisted of 380 (11.8%), 1,358 (42.1%), and 1,486 (46.1%) STEMI patients who underwent PCI, respectively. ROC curve analyses for each of the three groups (Supplementary Figure S2) showed that the AUC values for prediction of clinical outcomes were largest for the nighttime group (18:00–6:00) (AUC for mortality = 0.734, 95% CI: 0.677–0.791; AUC for MACEs = 0.707, 95% CI: 0.655–0.759); however, comparisons among the three groups indicated no statistically significant differences in the AUC values for in-hospital all-cause mortality (*P *= 0.614) or MACEs (*P *= 0.186). Finally, given that the 10-year span of the data collection period may have been a source of major heterogeneity, we performed ROC curve analyses with patients categorized into two 5-year subgroups, namely a 2010–2015 subgroup and a 2016–2020 subgroup (Supplementary Figure S3). These analyses revealed that the 10-year time heterogeneity was small, both in the case of in-hospital all-cause mortality and in the case of in-hospital MACEs (*P* for comparisons between AUCs of two subgroups = 0.583 and 0.446, respectively).

According to the restricted cubic spline model, as AAR increased, ORs for in-hospital all-cause mortality and MACEs increased (Supplementary Figure S4). Moreover, in order to confirm the predictive value of AAR for in-hospital all-cause mortality and MACEs, nomograms were constructed (Supplementary Figure S5). In summary, AAR provides satisfactory prognostic value in the prediction of adverse events during hospitalization.

### 3.4. AAR and long-term outcomes

**Figure 3:**
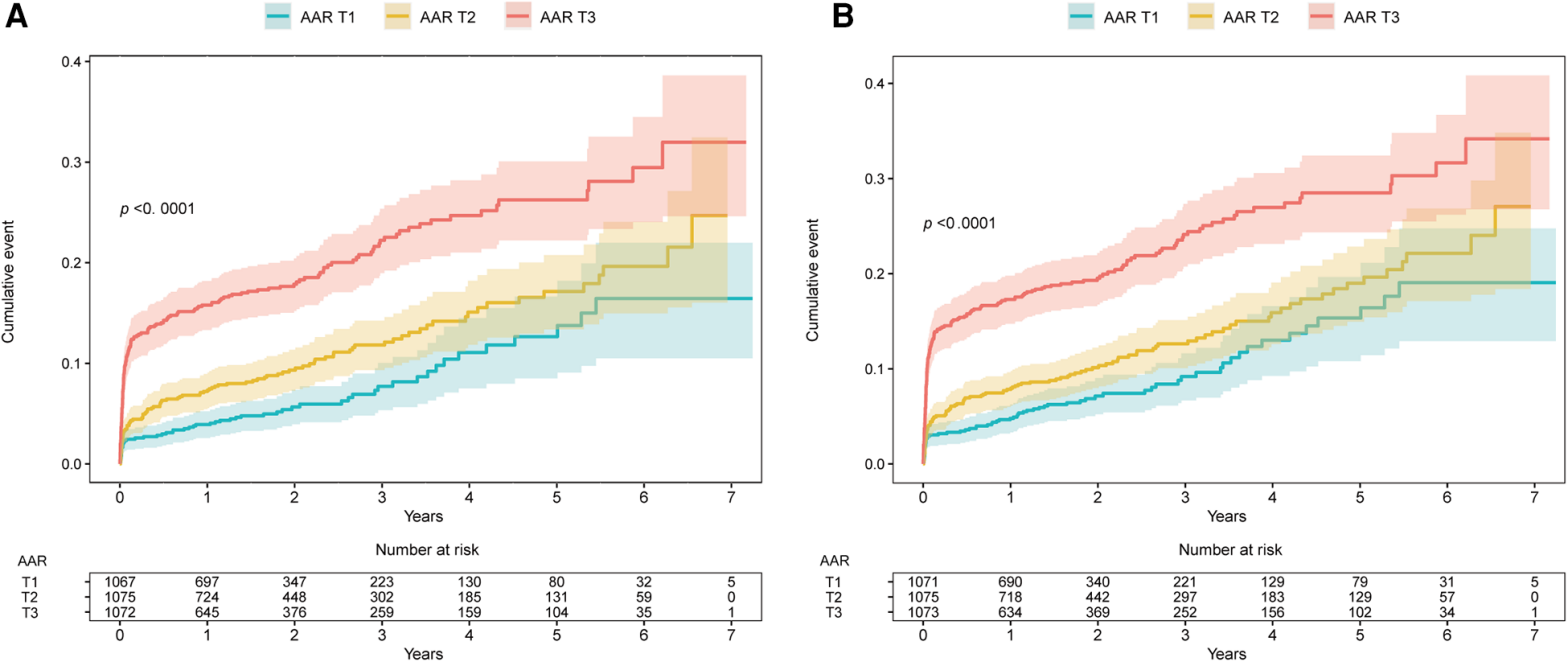
Kaplan–Meier survival curves for (A) long-term all-cause mortality and (B) long-term MACEs. AAR, admission-blood-glucose-to-albumin ratio; MACEs, major adverse cardiac events. AAR groups: T1, AAR value <3.35; T2, AAR value=3.35–4.56; T3, AAR value ≥4.56.

Over the median follow-up duration of 1.66 years, 339 cases of all-cause mortality and 376 MACEs occurred: by group, there were 57 (5.3%), 100 (9.3%), and 182 (16.9%) cases of all-cause mortality and 70 (6.5%), 108 (10.0%), and 198 (18.4%) MACEs in the T1 group (AAR < 3.35), the T2 group (3.35 ≤ AAR < 4.56), and the T3 group (AAR ≥ 4.56), respectively. Based on Kaplan–Meier curves, patients in the T3 group experienced higher rates of all-cause mortality and MACEs during follow-up than those in the T1 group (*P* < 0.0001) (Figure 3). According to the results of multivariable Cox regression analysis, AAR was found to be an independent risk factor for long-term all-cause mortality and MACEs, regardless of whether AAR was treated as a continuous variable or a categorical variable (Table 2 and Supplementary Table S3). In particular, when AAR was treated as a categorical variable, the T3 group had a higher risk of all-cause mortality [adjusted hazard ratio (HR): 1.64, 95% CI: 1.19–2.28, *P* = 0.003] as well as MACEs (adjusted HR: 1.58, 95% CI: 1.16–2.14, *P *= 0.003) compared with the T1 group (Table 2, Model 1). Similarly, the HRs for all-cause mortality and MACEs in different models were comparable when further adjusting for ALT (Table 2 and Supplementary Table S3, Model 2), insulin (Table 2 and Supplementary Table S3, Model 3), heart rate, and prior cardiac arrest (Table 2 and Supplementary Table S3, Model 4).

**Table 2:** Multivariable Cox regression analysis for all-cause mortality and MACEs during follow-up by AAR as a categorical variable.

AAR groups	All-cause mortality			MACEs		
	Adjusted HR	95% CI	*P*-value	Adjusted HR	95% CI	*P*-value
Model 1^a^						
T1		Ref.			Ref.	
T2	1.14	0.83–1.58	0.421	1.06	0.78–1.43	0.707
T3	1.64	1.19–2.28	0.003	1.58	1.16–2.14	0.003
Model 2^b^						
T1		Ref.			Ref.	
T2	1.13	0.81–1.58	0.470	1.06	0.78–1.45	0.710
T3	1.60	1.14–2.24	0.006	1.56	1.14–2.13	0.006
Model 3^c^						
T1		Ref.			Ref.	
T2	1.14	0.82–1.57	0.435	1.06	0.78–1.43	0.719
T3	1.60	1.15–2.22	0.005	1.54	1.14–2.10	0.006
Model 4^d^						
T1		Ref.			Ref.	
T2	1.09	0.79–1.50	0.615	1.01	0.75–1.37	0.924
T3	1.40	1.00–1.95	0.048	1.38	1.01–1.88	0.043

AAR, admission-blood-glucose-to-albumin ratio (AAR groups: T1, AAR < 3.35; T2, 3.35 ≤ AAR < 4.56; T3, AAR ≥ 4.56); CI, confidence interval; COPD, chronic obstructive pulmonary disease; DM, diabetes mellitus; eGFR, estimated glomerular filtration rate; HR, hazard ratio; MACEs, major adverse cardiac events; PCI, percutaneous coronary intervention.

^a^ Model 1: adjusted for age, sex, smoking status, prior stroke, COPD, DM, hypertension, prior myocardial infarction, prior PCI, anemia, Killip class ≥II, eGFR, aspirin, glycoprotein IIb/IIIa inhibitors, multiple lesions, and transradial access.

^b^ Model 2: adjusted for alanine aminotransferase (ALT) in addition to the adjustments in Model 1.

^c^ Model 3: adjusted for insulin in addition to the adjustments in Model 1.

^d^ Model 4: adjusted for heart rate and prior cardiac arrest in addition to the adjustments in Model 1.

## 4. Discussion

This is the first study to demonstrate the prognostic value of AAR for STEMI patients undergoing PCI. The results showed that there was an independent correlation between AAR and all-cause mortality and between AAR and MACEs during both hospitalization and follow-up. Furthermore, a cut-off value of AAR >4.429 had good discriminative ability for in-hospital all-cause mortality.

Rapid advances in imaging and laboratory examinations have improved our understanding of the etiology of STEMI. Inflammation is a key regulator of plaque, and an exposed lipid core triggers thrombosis. Thrombo-inflammatory reaction also occurs at the site of stent implantation after PCI (12). DM is a metabolic disorder with increased risks of myocardial infarction and consequent complications. In diabetic patients with STEMI, hyperglycemia might lead to oxidative stress, endothelial dysfunction, platelet activation, coagulation disturbances, and restenosis (3, 22, 23). In line with this, adverse events are more likely to occur in STEMI patients with elevated admission blood glucose at a level that is higher than normal but lower than DM thresholds (regarded as pre-diabetes) (24, 25). Therefore, admission blood glucose level may have prognostic value for adverse outcomes in STEMI patients undergoing PCI (4–6).

In STEMI patients, an elevated blood glucose level at admission indicates abnormal glucose metabolism due to chronic DM/pre-diabetes, the stress of the acute illness, or both. The association between chronic hyperglycemia (mainly attributed to DM) and adverse outcomes in STEMI patients is well known. In addition, SIH can worsen the cardiovascular outcomes of patients with or without DM, especially the latter group (9). A possible explanation is that SIH may exacerbate STEMI progression due to oxidative stress and tissue injury, while chronic hyperglycemia in DM may provoke a “preconditioning phenomenon” in cells and tissues, in response to the injury induced by SIH (9, 26). Roberts et al. (27) proposed the stress hyperglycemia ratio (SHR), consisting of corrected absolute blood glucose and HbA1c levels, as an index for the determination of background glycemia. It has been reported that the SHR can reflect relative glycemia and is independently related to the risks of MACEs and mortality in STEMI patients (28, 29). Nevertheless, the sensitivity of the SHR may be reduced in some patients with extremely low HbA1c levels, such as those with anemia or those who have recently undergone blood transfusion. Therefore, another biomarker is required to reflect the baseline status of patients.

Albumin, the most abundant plasma protein with highly susceptible targets for post-translational modifications, not only reflects aspects of malnutrition status (such as anemia), but is also related to DM because of the associated glycation (11, 30, 31). The progression of chronic DM leads to decreased plasma albumin levels because increased glycated albumin serves as a neo-epitope that disturbs the immune response and contributes to albuminuria. Furthermore, glycometabolism dysfunction hinders albumin synthesis (11). In addition, low albumin level is associated with increased glycated albumin and HbA1c levels in patients with DM (32, 33), some of whom may have experienced non-fatal myocardial infarction (33). Therefore, albumin serves as the denominator in the AAR index, in order to correct for malnutrition status and background glycemia.

Albumin, which is associated with STEMI progression and its treatment, has prognostic value for STEMI. Serum albumin is a negative acute-phase protein and provides antioxidant protection during inflammatory reactions. Furthermore, albumin exerts an anti-thrombotic effect via inhibition of platelet function and an anti-coagulant effect via neutralization of factor Xa, both of which are associated with the development of STEMI and with a hypercoagulable state after PCI (30, 31). Although chronic malnutrition diseases, such as cancer and liver or kidney diseases, reduce serum albumin levels, the low serum albumin level seen in patients with acute critical illnesses, such as STEMI, is caused by the increased catabolism and decreased synthesis of albumin. Several previous studies have reported that serum albumin has predictive value for STEMI, and decreased albumin level is associated with adverse outcomes after acute myocardial infarction. Serum albumin at admission is negatively correlated with risk of in-hospital mortality (13–16, 34), long-term mortality (16), and new-onset heart failure (13, 16) in STEMI patients. In addition, Kurtul et al. (35) reported that no-reflow after primary PCI is independently predicted by a low serum albumin level in STEMI patients. These associations may be attributed to the impact of albumin on ischemic events (36, 37). Therefore, the characteristics and functions of albumin can be combined with those of admission blood glucose to provide a composite biomarker for prediction of the risk of STEMI and subsequent reperfusion treatment.

Our results indicate that AAR, as a composite index based on admission blood glucose and albumin levels, has excellent prognostic value for STEMI patients undergoing PCI. In terms of comparison of the value of the composite of two indicators with that of a single indicator (Supplementary Figure S1), the results revealed that AAR was most favored in terms of evaluation of the likelihood of adverse events. Furthermore, we can observe the effects of these two separate indicators at work in this composite indicator: for one thing, the relatively stable albumin component of the AAR can indeed buffer the effect of admission blood glucose, whose level is relatively dynamic; for another, the effect of albumin may play a more critical role in AAR-based evaluation. Furthermore, time heterogeneity was also considered in our study. In particular, our results indicated that the predictive value of AAR was not disrupted either by the fluctuations in glucose levels that occurs over the course of a day or by time heterogeneity over the 10-year span of the study. Without doubt, when clinicians consider the role of nutrition, DM, and SIH in STEMI, the composite indicator AAR can help them to evaluate the status of a patient, because it reflects malnutrition, hyperglycemic status, and thrombo-inflammatory reaction during STEMI and PCI treatment. In addition, both admission blood glucose and albumin levels are cheap, easily available, and widely tested in the emergency department, meaning that measuring AAR is practicable in such acute conditions. In conclusion, the application of AAR is clinically feasible and may improve the determination of STEMI risk level at an early stage, thereby guiding STEMI treatment, particularly in developing countries.

The present study has some limitations. First, this was an observational study and therefore had inevitable bias and confounding factors. However, we attempted to minimize the bias during analysis. Second, we focused on AAR in the Chinese population. Other populations should be included in future studies. Third, we did not analyze levels of glycated albumin. Finally, considering the fact that plasma glucose levels shift dynamically over the course of a day while albumin is relatively stable over the same period, although we performed subgroup analyses with subgroups whose measurements were collected during different time periods, it would be better to monitor the dynamic glucose level of these patients. Therefore, further studies should be conducted to evaluate the effects of dynamic changes in plasma glucose on the predictive value of AAR for clinical outcomes.

## 5. Conclusion

In STEMI patients undergoing PCI, AAR is an independent predictor of all-cause mortality and MACEs both in hospital and during follow-up. AAR can be measured easily and rapidly for risk stratification, especially in primary hospitals.

## Data Availability

The raw data supporting the conclusions of this article will be made available by the authors, without undue reservation.
