# Supplementary material for: Association between admission-blood-glucose-to-albumin ratio and clinical outcomes in patients with ST-elevation myocardial infarction undergoing percutaneous coronary intervention
Source: Front Cardiovasc Med. 2023 Sep 7;10:1132685. doi: 10.3389/fcvm.2023.1132685 (PMC10513433; doi:10.3389/fcvm.2023.1132685)
Supplement: Supplementary file 1 [file Datasheet1.docx]

Supplementary Material

Association between admission blood glucose to albumin ratio and clinical outcomes in patients with ST-elevation myocardial infarction undergoing percutaneous coronary intervention

Cien Zhen, Wei Chen, Weikun Chen, Hualin Fan, Zijing Lin, Lihuan Zeng, Zehuo Lin, Weibin He, Yu Li, Shimin Peng, Lin Zeng, Chongyang Duan, Ning Tan, Yuanhui Liu*, Pengcheng He*.

*** Correspondence:** Pengcheng He: [gdhpc100@126.com](mailto:gdhpc100@126.com). Yuanhui Liu: liuyuanhui@gdph.org.cn.

# Supplementary Figure 1


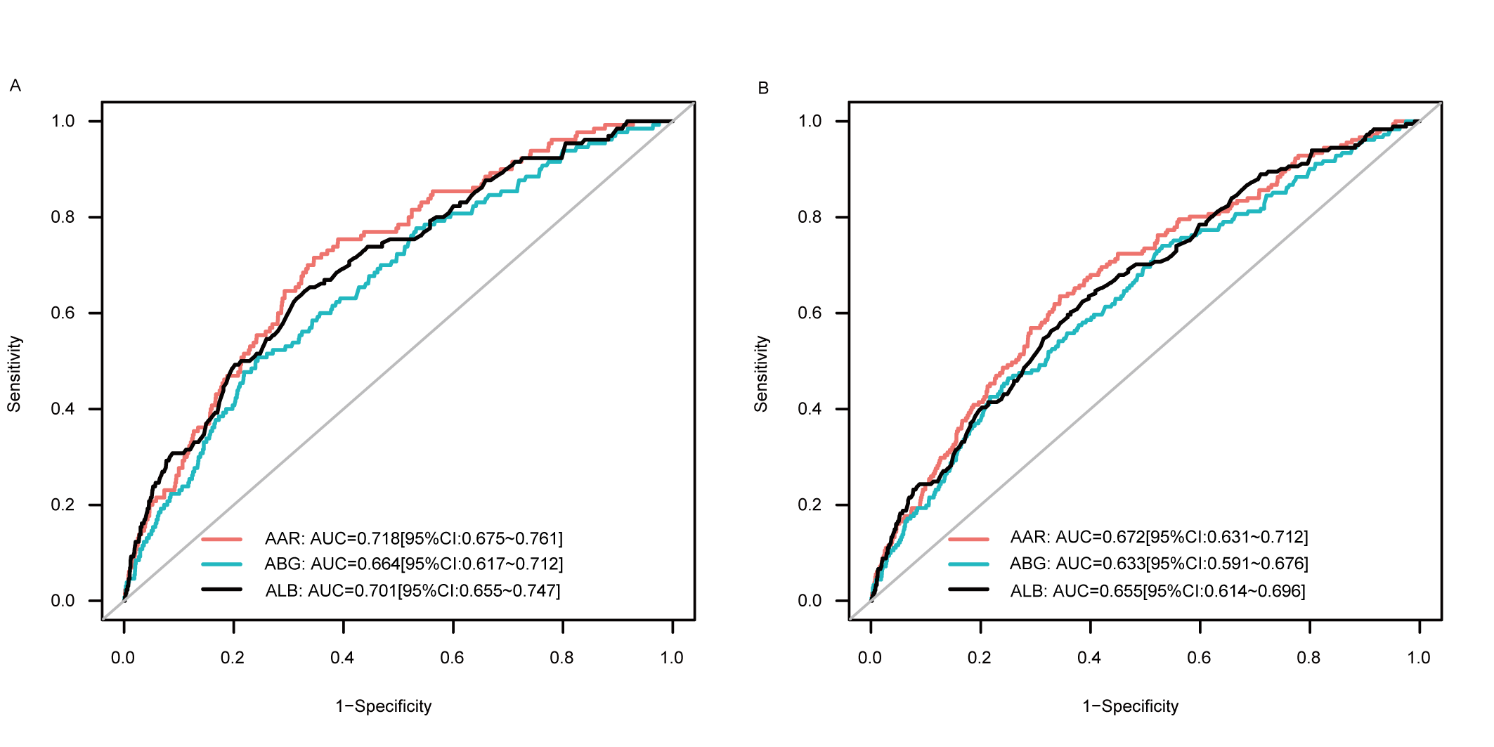


**Supplementary Figure 1** ROC curve of three indicators (including AAR, admission blood glucose and albumin) for in-hospital all-cause mortality and MACEs. (A) ROC curve of three indicators for in-hospital all-cause mortality; (B) ROC curve of three indicators for in-hospital MACEs. ROC, receiver operating characteristic; AUC, area under the ROC curve; AAR, admission blood glucose to albumin ratio; ABG, admission blood glucose; ALB, albumin; MACEs, major adverse cardiac events.

# Supplementary Figure 2


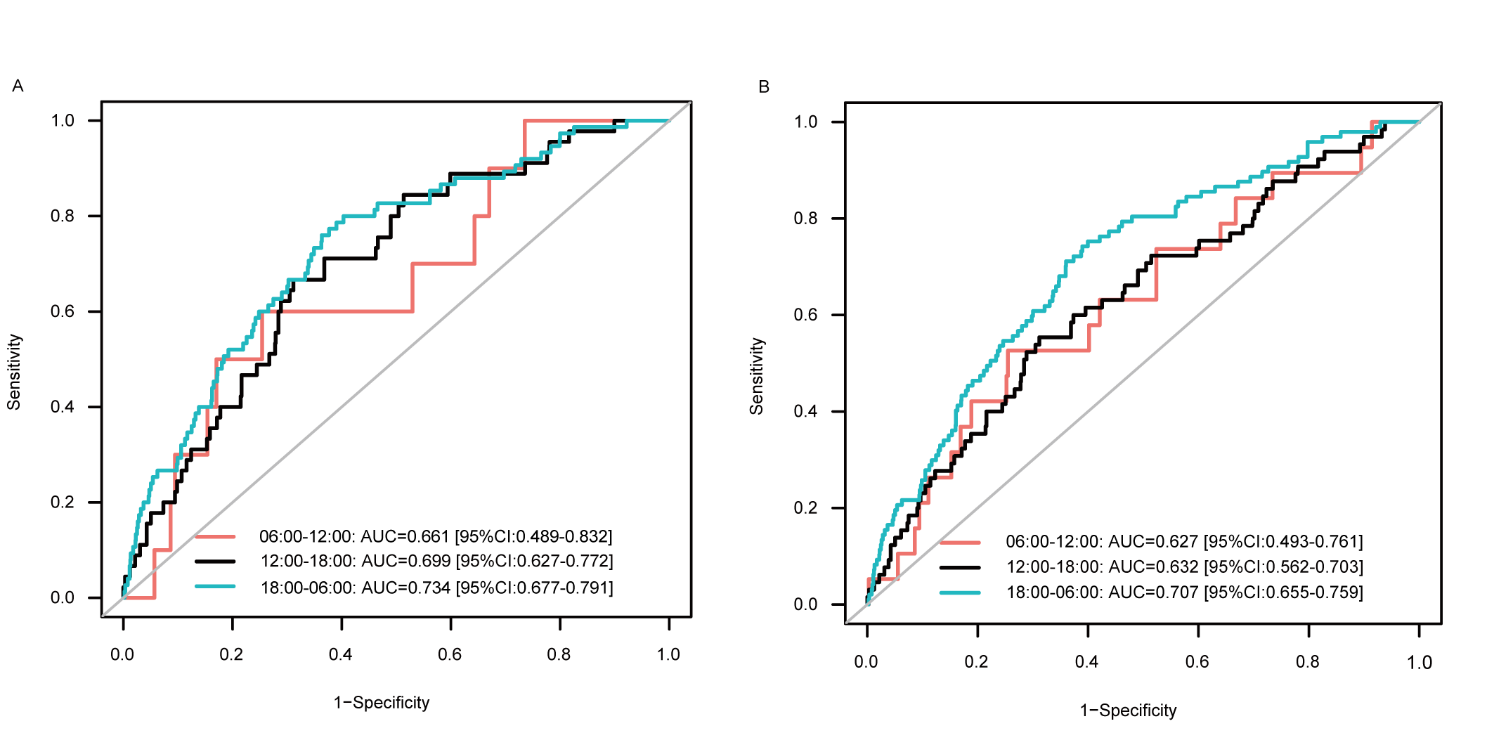


**Supplementary Figure 2** ROC curve of AAR for in-hospital all-cause mortality and MACEs in three time periods subgroup during one day [including 06:00-12:00 (morning), 12:00-18:00 (afternoon), 18:00-6:00 (night)]. (A) ROC curve of AAR for in-hospital all-cause mortality in three time periods subgroup; (B) ROC curve of AAR for in-hospital MACEs in three time periods subgroup. ROC, receiver operating characteristic; AUC, area under the ROC curve; AAR, admission blood glucose to albumin ratio; MACEs, major adverse cardiac events.

# Supplementary Figure 3


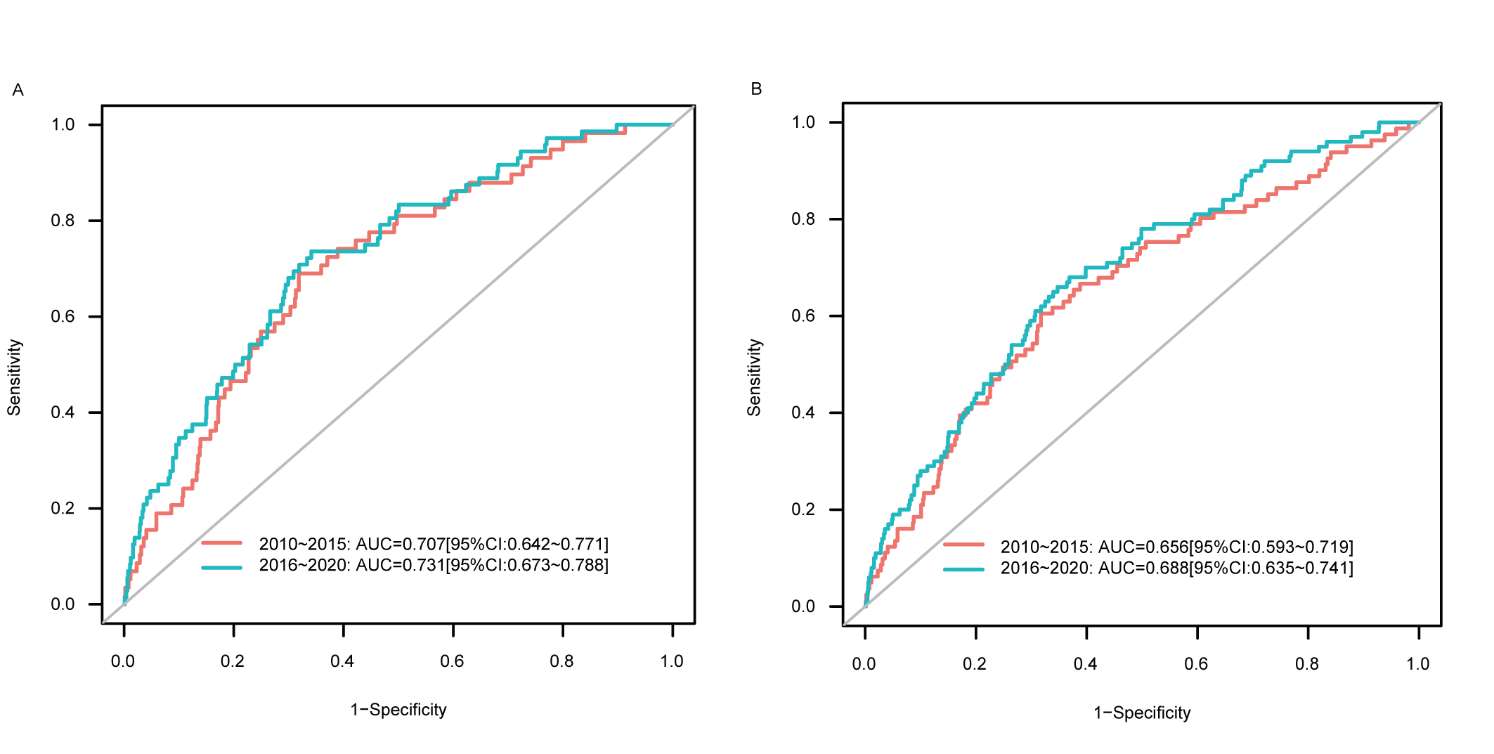


**Supplementary Figure 3** ROC curve of AAR for in-hospital all-cause mortality and MACEs in different five-year subgroup (including 2010-2015 subgroup and 2016-2020 subgroup). (A) ROC curve of AAR for in-hospital all-cause mortality in different five-year subgroup; (B) ROC curve of AAR for in-hospital MACEs in different five-year subgroup. ROC, receiver operating characteristic; AUC, area under the ROC curve; AAR, admission blood glucose to albumin ratio; MACEs, major adverse cardiac events.

# Supplementary Figure 4


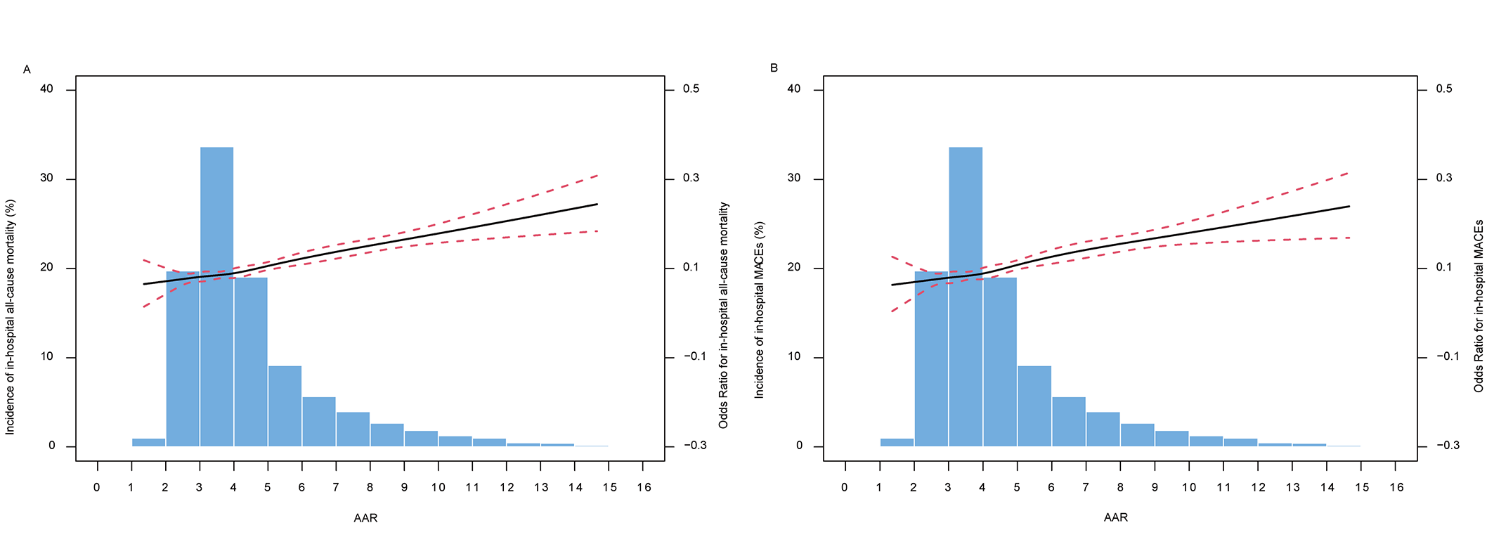


**Supplementary Figure 4** Nomograms predicting the risk of in-hospital all-cause mortality and MACEs. (A) Nomograms predicting the risk of in-hospital all-cause mortality; (B) Nomograms predicting the risk of in-hospital MACEs. MACEs, major adverse cardiac events; AAR, admission blood glucose to albumin ratio.

# Supplementary Figure 5

**
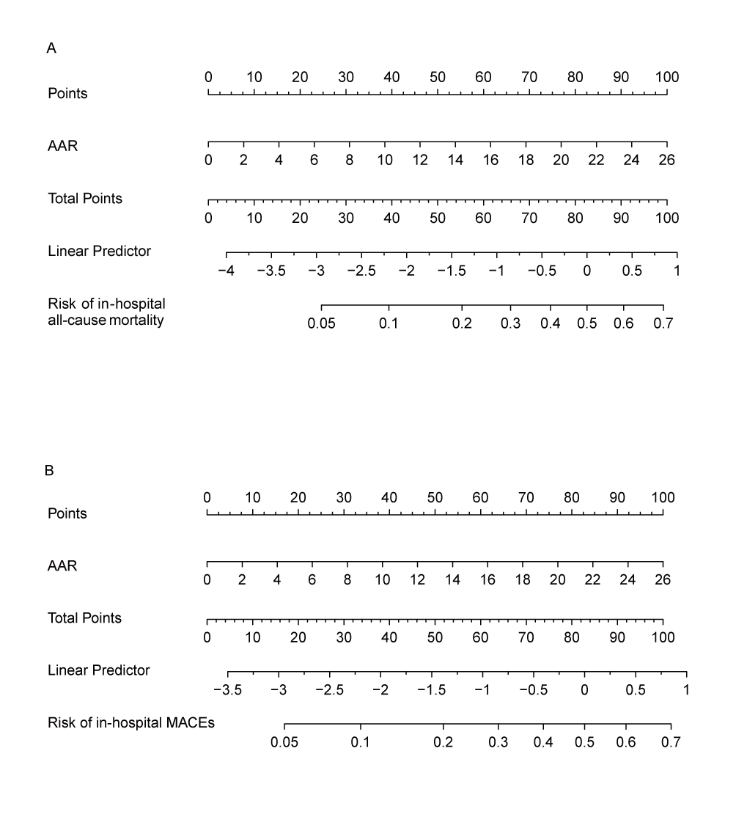
**

**Supplementary Figure 5** ORs for in-hospital all-cause mortality and MACEs by AAR according to restricted cubic spline model. (A) ORs for in-hospital all-cause mortality and MACEs by AAR level; (B) ORs for in-hospital MACEs by AAR level. OR, odds ratio; MACEs, major adverse cardiac events; AAR, admission blood glucose to albumin ratio.

# Supplementary Table 1 Multivariable Logistic regression analysis of risk factors for in-hospital all-cause mortality and in-hospital MACEs according to AAR as the continuous variables.

|  | In-hospital all-cause mortality | | |  | In-hospital MACEs | | | | |
| --- | --- | --- | --- | --- | --- | --- | --- | --- | --- |
|  | Adjusted OR | 95% CI | *P*-value |  | Adjusted OR | | 95% CI | | *P*-value |
| AAR, per 1-unit increase |  |  |  |  |  |  | |  | |
| Model 1**^†^** | 1.14 | 1.07 - 1.22 | 0.000 |  | 1.11 | 1.05 - 1.18 | | 0.001 | |
| Model 2**^‡^** | 1.12 | 1.04 - 1.20 | 0.001 |  | 1.09 | 1.02 - 1.16 | | 0.007 | |
| Model 3**^§^** | 1.14 | 1.06 - 1.22 | 0.000 |  | 1.11 | 1.04 - 1.18 | | 0.001 | |
| Model 4**^*^** | 1.13 | 1.06 - 1.21 | 0.000 |  | 1.10 | 1.04 - 1.17 | | 0.002 | |

Legends: MACEs, major adverse cardiac events; AAR, admission blood glucose to albumin ratio; OR, odds ratio; CI, confidence interval.

**^†^** Model 1: adjusted for age, female sex, smoking, prior stroke, chronic obstructive pulmonary disease, diabetes mellitus (DM), hypertension, prior myocardial infarction, prior PCI, anemia, Killip class ≥ II, estimated glomerular filtration rate (eGFR), aspirin, glycoprotein IIb/IIIa inhibitors, multiple lesions, transradial access;

**^‡^** Model 2: adjusted for alanine aminotransferase (ALT) in addition to Model 1;

**^§^** Model 3: adjusted for insulin in addition to Model 1;

**^*^** Model 4: adjusted for heart rate and prior cardiac arrest in addition to Model 1.

# Supplementary Table 2 Multivariable Logistic regression analysis for in-hospital all-cause mortality and in-hospital MACEs according to AAR as the categorical variables.

| AAR groups | In-hospital all-cause mortality | | |  | In-hospital MACEs | | |
| --- | --- | --- | --- | --- | --- | --- | --- |
|  | Adjusted OR | 95% CI | *P*-value |  | Adjusted OR | 95% CI | *P*-value |
| Model 1**^†^** |  |  |  |  |  |  |  |
| T1 |  | Ref. |  |  |  | Ref. |  |
| T2 | 1.10 | 0.58-2.08 | 0.782 |  | 0.94 | 0.57-1.52 | 0.788 |
| T3 | 2.72 | 1.47-5.03 | 0.001 |  | 1.91 | 1.18-3.10 | 0.009 |
| Model 2**^‡^** |  |  |  |  |  |  |  |
| T1 |  | Ref. |  |  |  | Ref. |  |
| T2 | 1.18 | 0.60 - 2.33 | 0.630 |  | 0.97 | 0.58 - 1.60 | 0.895 |
| T3 | 2.96 | 1.55 - 5.65 | 0.001 |  | 1.95 | 1.18 - 3.20 | 0.009 |
| Model 3**^§^** |  |  |  |  |  |  |  |
| T1 |  | Ref. |  |  |  | Ref. |  |
| T2 | 1.09 | 0.57 - 2.08 | 0.789 |  | 0.93 | 0.57 - 1.52 | 0.781 |
| T3 | 2.67 | 1.44 - 4.96 | 0.002 |  | 1.86 | 1.15 - 3.04 | 0.012 |
| Model 4**^*^** |  |  |  |  |  |  |  |
| T1 |  | Ref. |  |  |  | Ref. |  |
| T2 | 1.02 | 0.54 - 1.95 | 0.944 |  | 0.89 | 0.54 - 1.46 | 0.644 |
| T3 | 2.24 | 1.20 - 4.18 | 0.011 |  | 1.64 | 1.00 - 2.67 | 0.049 |

Legends: MACEs, major adverse cardiac events; AAR, admission blood glucose to albumin ratio; OR, odds ratio; CI, confidence interval; AAR groups: T1, AAR < 3.35; T2, 3.35 ≤ AAR < 4.56; T3, AAR ≥ 4.56.

**^†^** Model 1: adjusted for age, female sex, smoking, prior stroke, chronic obstructive pulmonary disease, diabetes mellitus (DM), hypertension, prior myocardial infarction, prior PCI, anemia, Killip class ≥ II, estimated glomerular filtration rate (eGFR), aspirin, glycoprotein IIb/IIIa inhibitors, multiple lesions, transradial access;

**^‡^** Model 2: adjusted for alanine aminotransferase (ALT) in addition to Model 1;

**^§^** Model 3: adjusted for insulin in addition to Model 1;

**^*^** Model 4: adjusted for heart rate and prior cardiac arrest in addition to Model 1.

# Supplementary Table 3 Multivariable Cox regression analysis for all-cause mortality and MACEs during following up according to AAR as the continuous variables.

|  | All-cause mortality | | |  | MACEs | | | | |
| --- | --- | --- | --- | --- | --- | --- | --- | --- | --- |
|  | Adjusted HR | 95% CI | *P*-value |  | Adjusted OR | | 95% CI | | *P*-value |
| AAR, per 1-unit increase |  |  |  |  |  |  | |  | |
| Model 1**^†^** | 1.09 | 1.06 - 1.13 | <0.001 |  | 1.09 | 1.05 - 1.13 | | <0.001 | |
| Model 2**^‡^** | 1.08 | 1.04 - 1.12 | <0.001 |  | 1.08 | 1.04 - 1.12 | | <0.001 | |
| Model 3**^§^** | 1.09 | 1.05 - 1.13 | <0.001 |  | 1.09 | 1.05 - 1.13 | | <0.001 | |
| Model 4**^*^** | 1.08 | 1.04 - 1.13 | <0.001 |  | 1.08 | 1.04 - 1.12 | | <0.001 | |

Legends: MACEs, major adverse cardiac events; AAR, admission blood glucose to albumin ratio; HR, hazard ratio; CI, confidence interval.

**^†^** Model 1: adjusted for age, female sex, smoking, prior stroke, chronic obstructive pulmonary disease, diabetes mellitus (DM), hypertension, prior myocardial infarction, prior PCI, anemia, Killip class ≥ II, estimated glomerular filtration rate (eGFR), aspirin, glycoprotein IIb/IIIa inhibitors, multiple lesions, transradial access;

**^‡^** Model 2: adjusted for alanine aminotransferase (ALT) in addition to Model 1;

**^§^** Model 3: adjusted for insulin in addition to Model 1;

**^*^** Model 4: adjusted for heart rate and prior cardiac arrest in addition to Model 1.
