# Supplementary figures and images for: Association between admission-blood-glucose-to-albumin ratio and clinical outcomes in patients with ST-elevation myocardial infarction undergoing percutaneous coronary intervention
Source: Front Cardiovasc Med. 2023 Sep 7;10:1132685. doi: 10.3389/fcvm.2023.1132685 (PMC10513433; doi:10.3389/fcvm.2023.1132685)

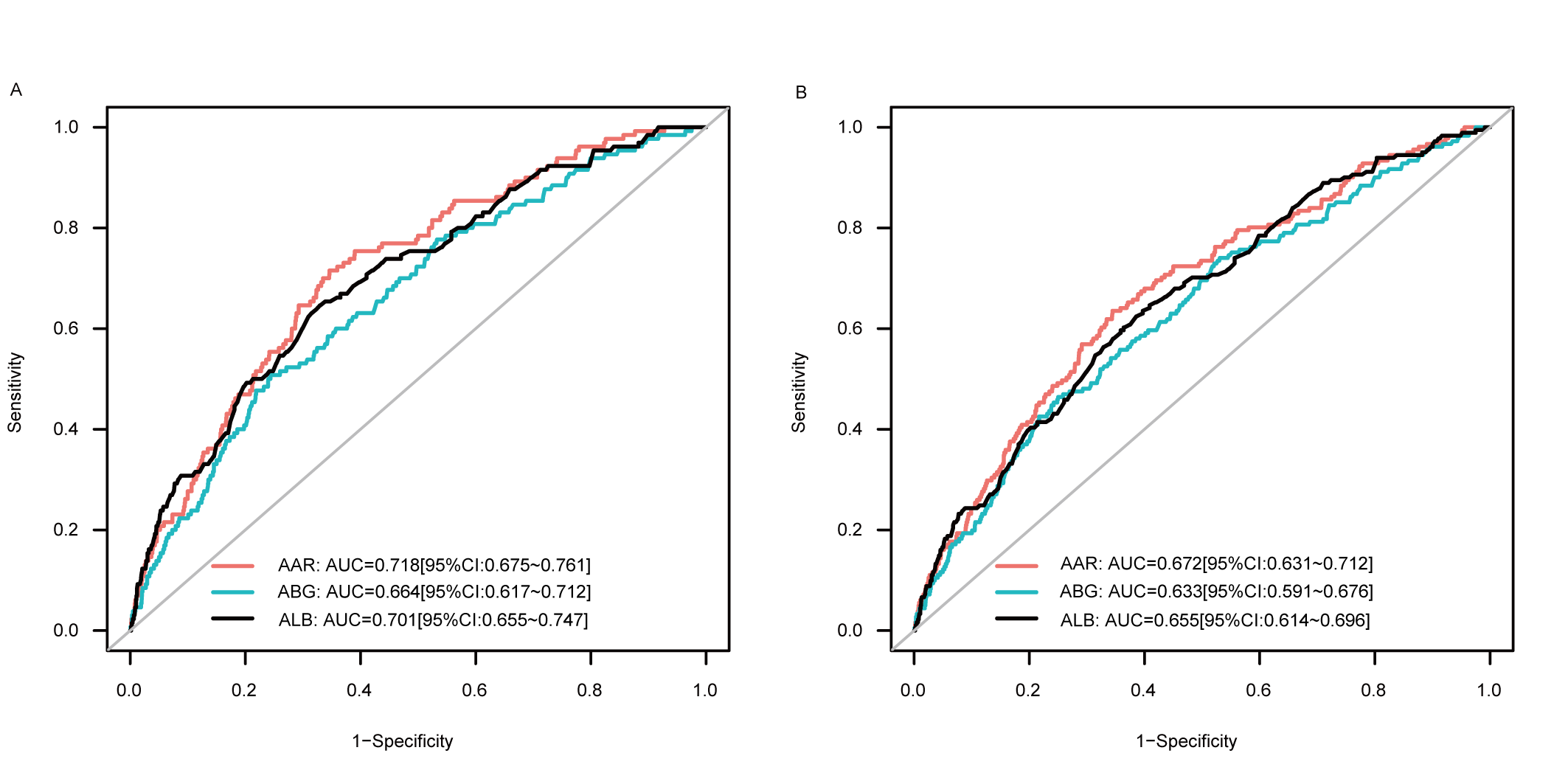

Supplement: Supplementary file 2 [file Image1.tif]

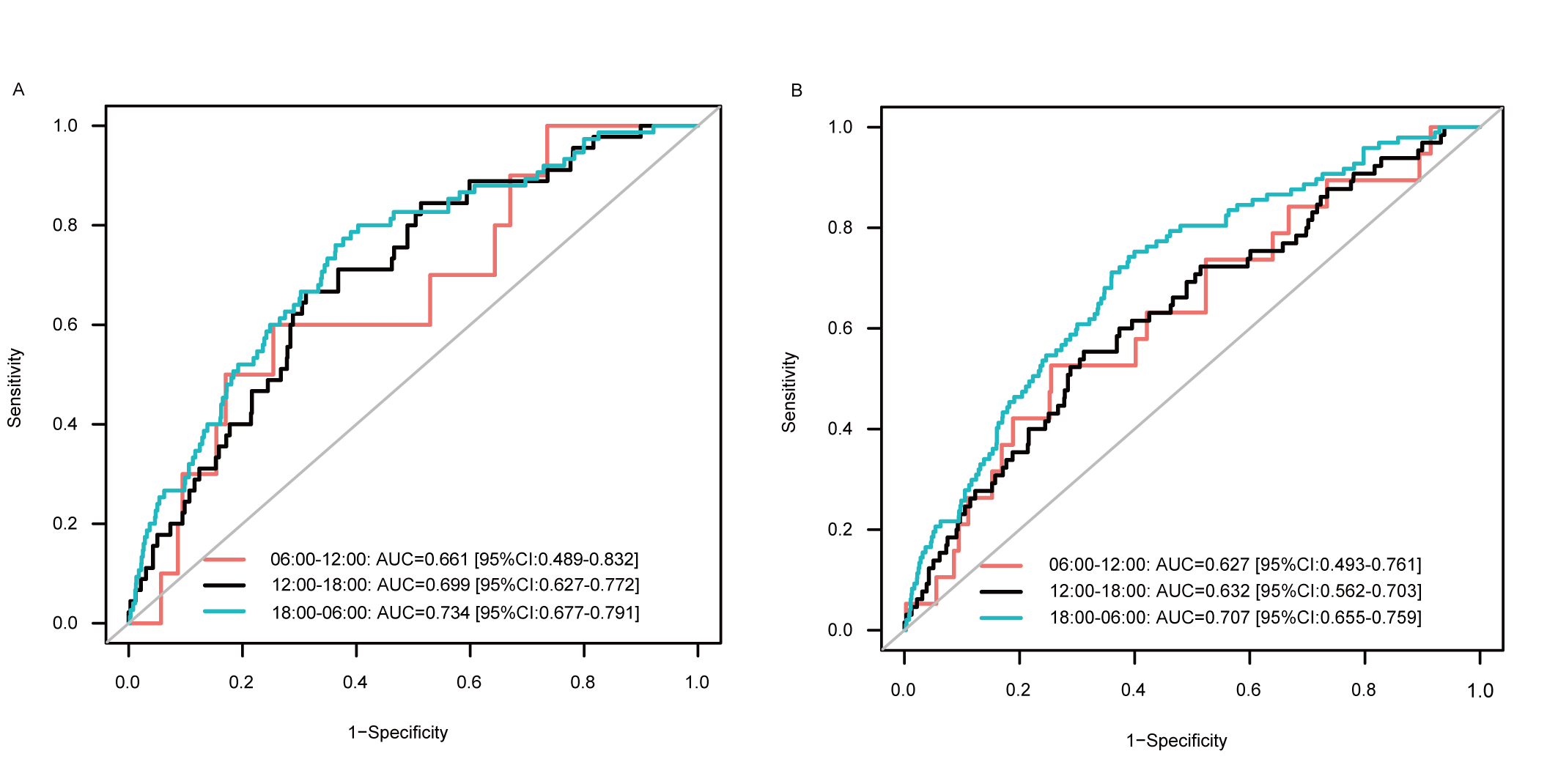

Supplement: Supplementary file 3 [file Image2.tif]

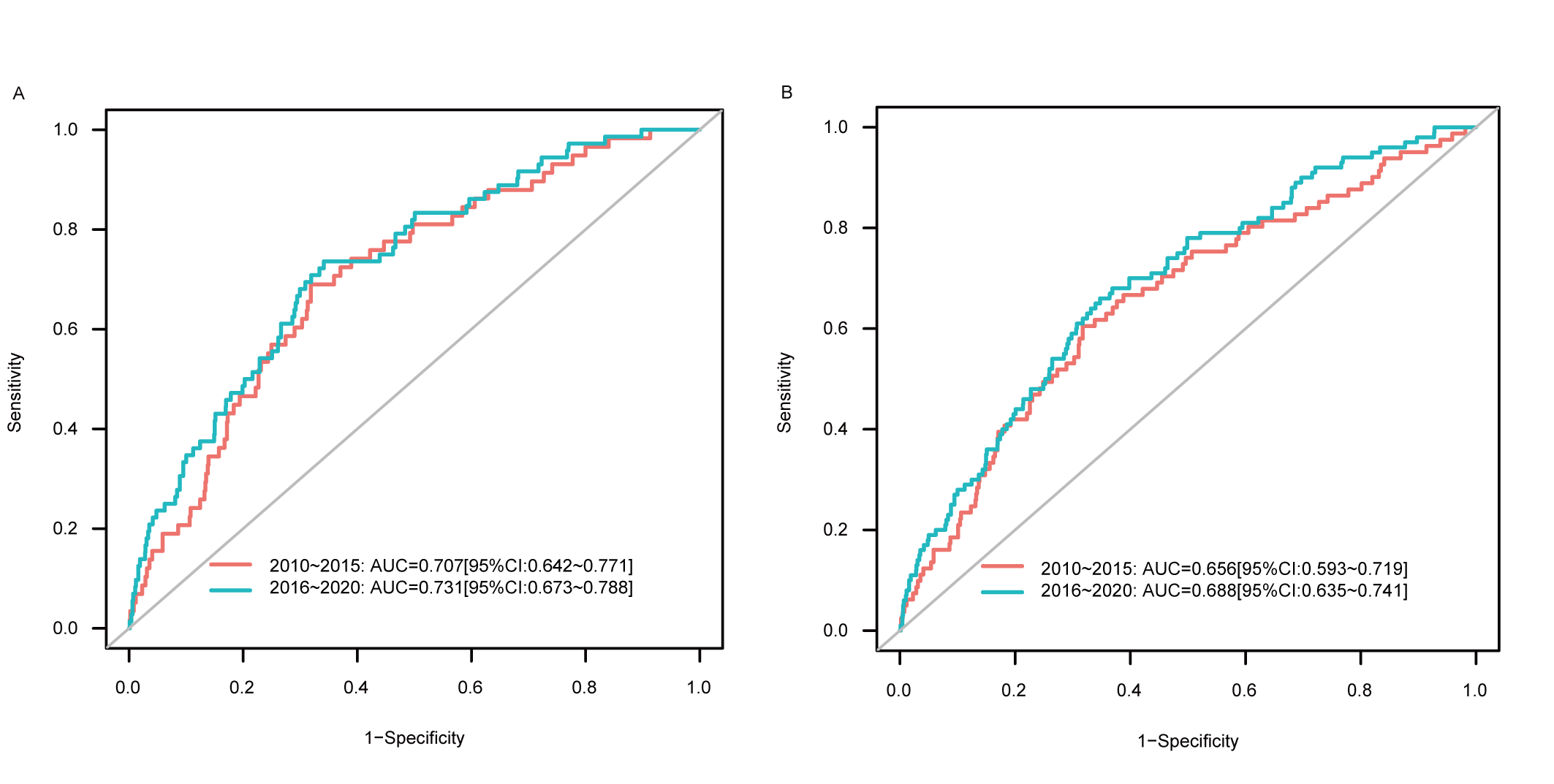

Supplement: Supplementary file 4 [file Image3.tif]

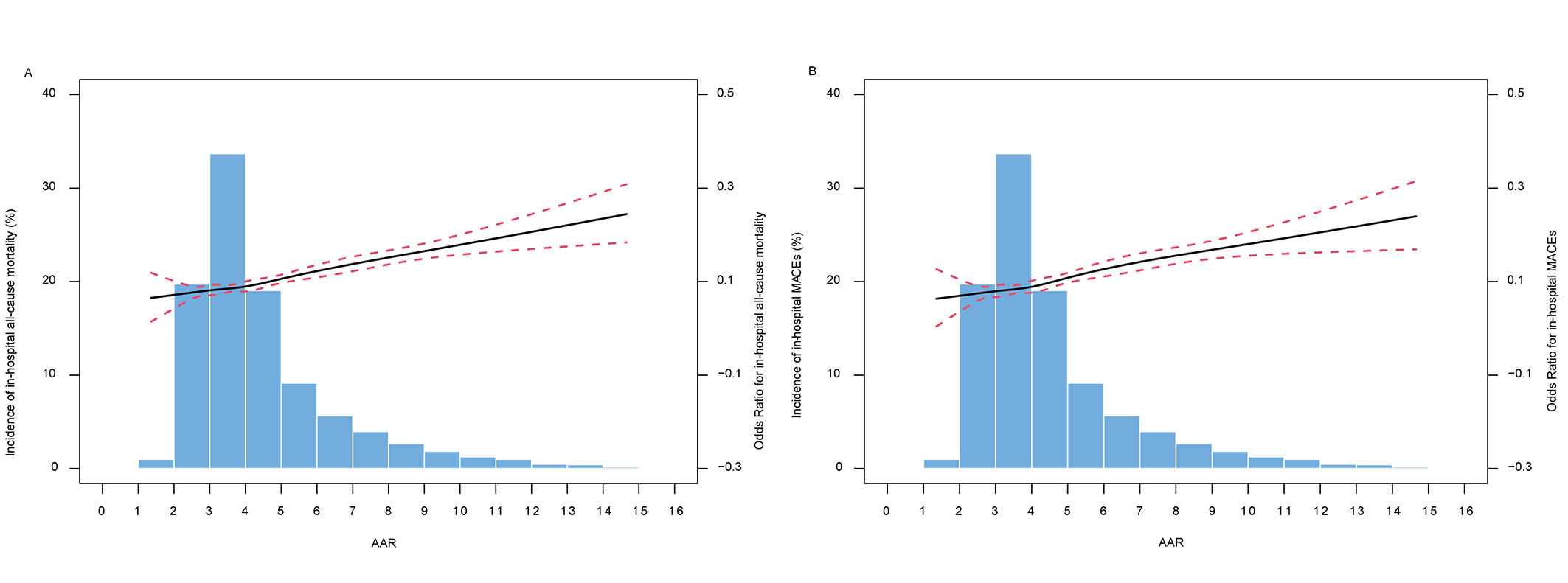

Supplement: Supplementary file 5 [file Image4.tif]

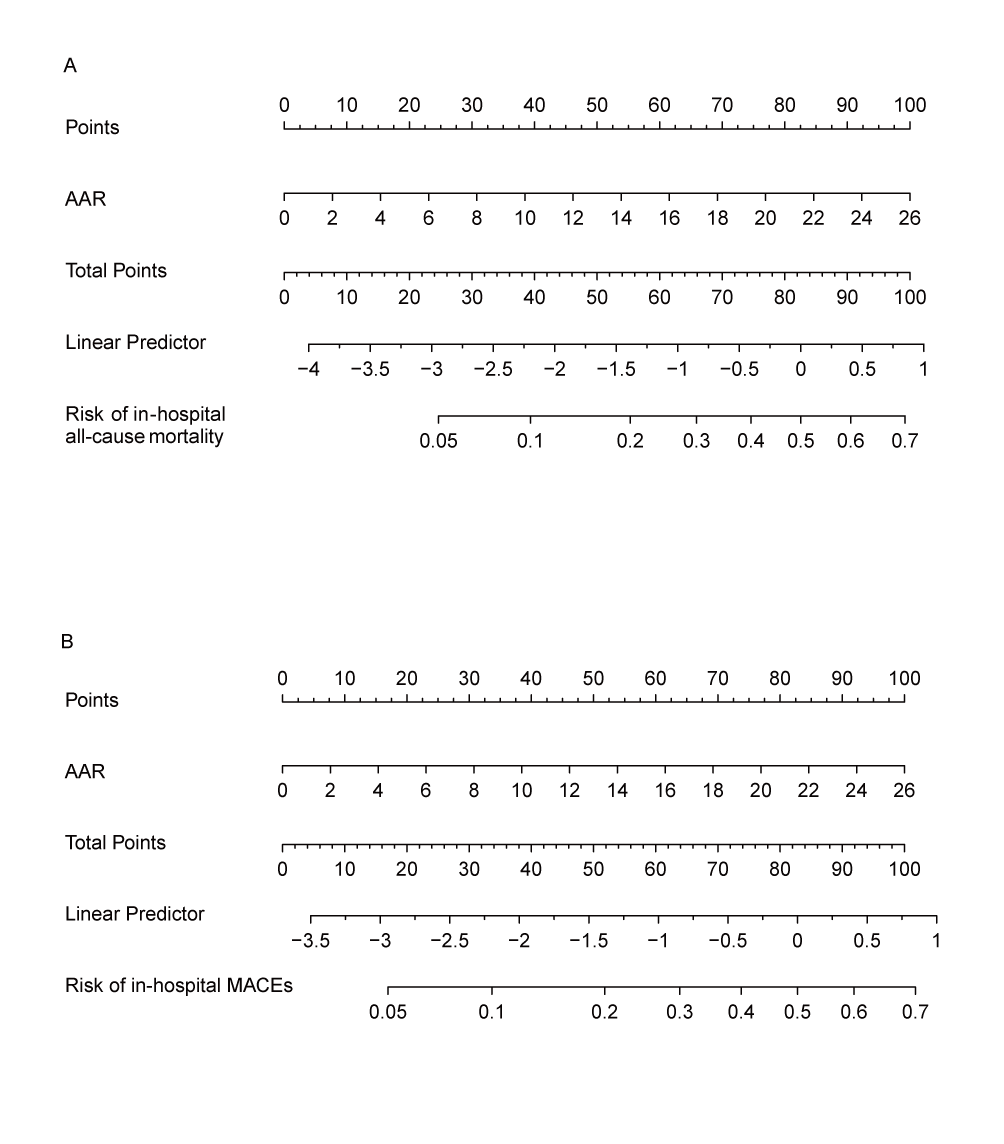

Supplement: Supplementary file 6 [file Image5.tif]
